# Exosomes mediate an epithelial‐mesenchymal transition cascade in retinal pigment epithelial cells: Implications for proliferative vitreoretinopathy

**DOI:** 10.1111/jcmm.15951

**Published:** 2020-10-13

**Authors:** Yao Zhang, Kaizhe Wang, Jiabin Pan, Shuai Yang, Haipei Yao, Min Li, Hui Li, Hetian Lei, Haiying Jin, Fang Wang

**Affiliations:** ^1^ Department of Ophthalmology Shanghai Tenth People's Hospital Tongji University School of Medicine Shanghai China; ^2^ Division of Physical Biology and Bioimaging Center Shanghai Synchrotron Radiation Facility CAS Key Laboratory of Interfacial Physics and Technology Shanghai Institute of Applied Physics Chinese Academy of Sciences Shanghai China; ^3^ Center for Translational Neurodegeneration and Regenerative Therapy Shanghai Tenth People's Hospital Tongji University School of Medicine Shanghai China; ^4^ Department of Ophthalmology Schepens Eye Research Institute of Massachusetts Eye and Ear Harvard Medical School Boston MA USA

**Keywords:** epithelial‐mesenchymal transition, proliferative vitreoretinopathy, retinal pigment epithelium

## Abstract

Exosomes have recently emerged as a pivotal mediator of many physiological and pathological processes. However, the role of exosomes in proliferative vitreoretinopathy (PVR) has not been reported. In this study, we aimed to investigate the role of exosomes in PVR. Transforming growth factor beta 2 (TGFß‐2) was used to induce epithelial‐mesenchymal transition (EMT) of retinal pigment epithelial (RPE) cells, as an in vitro model of PVR. Exosomes from normal and EMTed RPE cells were extracted and identified. We incubated extracted exosomes with recipient RPE cells, and co‐cultured EMTed RPE cells and recipient RPE cells in the presence of the exosome inhibitor GW4869. Both experiments suggested that there are further EMT‐promoting effects of exosomes from EMTed RPE cells. MicroRNA sequencing was also performed to identify the miRNA profiles in exosomes from both groups. We identified 34 differentially expressed exosomal miRNAs (*P* <. 05). Importantly, miR‐543 was found in exosomes from EMTed RPE cells, and miR‐543‐enriched exosomes significantly induced the EMT of recipient RPE cells. Our study demonstrates that exosomal miRNA is differentially expressed in RPE cells during EMT and that these exosomal miRNAs may play pivotal roles in EMT induction. Our results highlight the importance of exosomes as cellular communicators within the microenvironment of PVR.

## 1 INTRODUCTION

Proliferative vitreoretinopathy (PVR) is characterized by the formation of subretinal, epiretinal and intravitreal fibrotic membranes that can lead to tractional retinal detachment, and usually occurs after rhegomatogenous retinal detachment (RRD) and its surgical treatment.^1^, ^2^ Pathological studies suggest that an excessive wound healing response including cellular proliferation and migration and extracellular matrix production, and remodelling participates in the formation of PVR membranes. Among the many cellular events that occur in PVR, the transdifferentiation of retinal pigment epithelial (RPE) cells into mesenchymal cells via epithelial‐mesenchymal transition (EMT) has been regarded as the trigger in PVR pathogenesis.^3^, ^4^, ^5^ The retinal pigment epithelium, located between the neural retina and Bruch's membrane, is a monolayer of highly polarized epithelial cells. EMT enables RPE cells to lose epithelial properties, transdifferentiate into mesenchymal cells, migrate into vitreous cavity and proliferate into membranes. Ultimately, contraction of these membranes leads to structural and functional damage to the retina.^5^

Exosomes are nano‐sized membrane vesicles (30‐150 nm in diameter) secreted by a range of cells.^6^, ^7^ Exosomes contain microRNA (miRNAs), mRNA, DNA, cytoplasmic proteins and lipids. These components can deliver specific molecular messages and cause various responses in recipient cells.^8^ It has been shown that exosomes play a fundamental role in the regulation of physiological situations, as well as in pathological processes including central nervous system diseases, myocardial ischaemia/circulation damage, liver and kidney injuries, and the modulation of tumour angiogenesis and metastasis.^9^ Recent studies have suggested that exosomes can induce EMT in recipient cells. Luga et al^10^ observed that exosomes derived from cancer‐associated fibroblasts (CAFs) promoted EMT in recipient breast cancer cells via activating autocrine WNT‐planar cell polarity signalling. In melanoma, exosomal WNT not only promoted EMT in recipient cells, but also changed the composition of released exosomes to promote further EMT.^11^ Another study conducted by Koch et al^12^ suggested that diffuse large B‐cell lymphomas possess a self‐organized infrastructure comprising two populations of cells, where transitions between clonogenic states can be modulated by exosome‐mediated WNT signalling.

MicroRNAs are noncoding RNAs that average 22 nucleotides in length. They repress target mRNAs, regulate gene expression^13^, ^14^, ^15^ and participate in many biological processes.^16^ In addition to their endogenous actions, miRNAs can be secreted into the extracellular space within exosomes.^17^, ^18^, ^19^ Cell‐derived exosomes contain many miRNAs, and these exosomes can be taken up into neighbouring or distant cells to modulate the function of the recipient cell.^20^, ^21^, ^22^ In recent years, exosomal miRNAs have received increased attention, particularly in tumour microenvironment research. Li et al found that CAFs contribute to cancer cell proliferation and metastasis via exosomal miR‐34a‐5p.^23^ Another study conducted by Wang et al suggest that exosomal delivery of miR‐155‐5p could promote EMT and chemoresistance in gastric cancer cells.^24^ Exosomal miR‐32‐5p has also been reported to play a role in multidrug resistance in hepatocellular carcinoma.^25^

With the delivery of their contents, exosomes can exert precise effects on cellular interactions within the local microenvironment, as well as on signal spreading for distant cellular communications. In carcinogenesis, the roles of exosomes in both tumour microenvironments and metastasis have been well studied. However, the effect of exosomes on EMT of RPE cells in the vitreoretinal microenvironment and PVR has not yet been investigated. In this study, we examined the effects of transitioning from the epithelial state to the mesenchymal state on the release and contents of exosomes in RPE cells. Furthermore, we investigated the role of exosomes from EMTed RPE cells on recipient RPE cells.

## 2 MATERIALS AND METHODS

### 2.1 Reagents and antibodies

Human recombinant transforming growth factor beta 2 (TGFβ‐2) was purchased from R&D (Minneapolis, MN, USA). ExoQuick‐TC Exosome Precipitation Solution, SeraMir Exosome RNA Amplification and Exo‐Fect Transfection Kits were purchased from SBI (EXOTC50A‐1, RA806TC‐1, EXFT20A‐1, System Biosciences, Palo Alto, CA, USA). miDETECT A Track™ miRNA qRT‐PCR Starter Kit and primers for miRNA detection were purchased from RiboBio. The following antibodies were used for Western blotting and immunofluorescence: α‐smooth muscle actin (A2547 Sigma‐Aldrich, St.Louis, MO, USA) and fibronectin (F7387; Sigma‐Aldrich, St.Louis, MO, USA), E‐cadherin (610182 BD Biosciences, San Jose, CA, USA), β‐actin (ab119716; Abcam Ltd., Cambridge, MA, USA) and Alix (BS70704; Bioworld Technology, Inc) CD63, CD81, and CD9 (System Biosciences). Secondary antibodies for Western blots included IRDye 800CW and IRD 680LT (Li‐Cor Biosciences, Lincoln, NE, USA). Most other reagents such as salt and buffer components were analytical grade and obtained from Sigma‐Aldrich.

### 2.2 Cell culture

ARPE‐19 cells were cultured at 37°C in a 5% CO_2_ humidified incubator with a 1:1 mixture of Dulbecco's modified eagle's medium and Ham's F12 medium (DMEM/F12; Gibco, Carlsbad, CA, USA) supplemented with 10% foetal bovine serum (FBS; Gibco) and 1% Penicillin‐Streptomycin. The medium was changed every 2‐3 days.

### 2.3 Transwell co‐culture assay

Co‐culture experiments were carried out via a transwell co‐culture system. Normal ARPE‐19 cells, EMTed ARPE‐19 cells, and EMTed ARPE‐19 cells with GW4869 were seeded in the lower compartments of 24‐well transwell plates (Costar Corning, CA, USA) with 600 μL of serum‐free DMEM/F12 medium. Recipient ARPE‐19 cells were seeded in the upper chamber in 100 μL of serum‐free DMEM/F12 medium. The chambers were then incubated at 37°C for 48 hours, and the EMT markers of recipient ARPE‐19 cells were detected by Western blot, qPCR and immunofluorescence.

### 2.4 Exosome isolation

Exosomes were collected using the ExoQuick (System Biosciences) precipitation method. In brief, cell supernatants were collected and were centrifuged at 3000 g for 15 minutes to remove cell debris. ExoQuick Solution was then added to the supernatants (1:5), mixed well and incubated overnight at 4°C. After incubation, the mixture was centrifuged at 1500 g for 30 minutes, and the supernatant was removed. Exosome pellets were suspended in PBS and passed through a 0.22‐μm filter. The characterization of exosomes was confirmed by nanoparticle tracking analysis (NTA), transmission electron microscopy (TEM) analysis and Western blotting.

### 2.5 Transmission electron microscopy

Exosomes were analysed using TEM. We fixed 20 μL of exosome suspension (5 µg/µL) on a continuous grid, negatively stained with 2% uranyl acetate solution for 1 minute and air‐dried. The samples were observed using a FEI Tecnai G2 spirit transmission electron microscope (FEITM, Hillsboro, OR, USA) at an acceleration voltage of 120 kV.

### 2.6 Nanoparticle tracking analysis

Nanoparticle tracking analysis (NTA) measurements were performed using a NanoSight NS300 instrument (Malvern Panalytical, Malvern, UK). ARPE‐19 cells and EMTed ARPE‐19 cells were cultured in 10 cm culture dishes. Exosomes were isolated from normalized volumes of serum‐free culture supernatant through the ExoQuick Kit and resuspended with PBS. The supernatant was diluted at 1:100 in PBS, and 1 mL of solution was used for NTA analysis.

### 2.7 Exosome uptake analysis

Exosomes isolated from both normal and EMTed ARPE‐19 cells were labelled with CM‐Dil membrane dye (Thermo Fisher Scientific, Waltham, MA, USA) as follows: briefly, exosomes were mixed with CM‐Dil (1 μmol/L). A mixture without exosomes was used as a negative control. The mixture was incubated for 5 minutes at 37°C, and the exosomes were then harvested using ExoQuick‐TC. The pellets were resuspended in 1 mL of DMEM/F12 with 1% bovine serum albumin (BSA). ARPE‐19 cells were stained with PKH67 (Millipore‐Sigma, Burlington, MA, USA) according to the manufacture's protocol and then incubated with labelled exosomes for 24 hours at 37°C. After incubation, the cells were washed twice with PBS and fixed with 4% paraformaldehyde in PBS for 30 minutes at room temperature. Nuclei were stained with DAPI. The cellular uptake of exosomes was examined by confocal microscopy (TCS SP8; Leica, Wetzlar, Germany).

### 2.8 miRNA library construction and sequencing

Total RNA from exosomes was used for miRNA library preparation and sequencing. Library preparation and sequencing were performed at RiboBio. Briefly, total RNA samples were fractionated on a 15% Tris‐borate‐EDTA (TBE) polyacrylamide gel and small RNAs ranging between 18 and 30 nucleotides in size were used for library preparation. Small RNAs were reverse transcribed and amplified by PCR. The PCR products were sequenced using an Illumina HiSeq 2500 platform.

### 2.9 Exosome transfection

Exosome transfection was performed using an Exo‐Fect Transfection Kit. Briefly, miR543 mimics/MOCK miR was incubated with Exo‐Fect transfection reagent for 15 minutes at RT and exosomes from normal ARPE‐19 cells were added to the mixture and incubated at 37°C for 1 hour. The reaction was transferred to the pre‐washed spin‐column and incubated with gentle rotation for 10 minutes at RT. Exosomes loaded with miR543/MOCK miR were collected by centrifuging the spin‐column for 30 seconds at 1000 g.

### 2.10 Real‐time quantitative PCR

Total RNA was extracted at the indicated timepoints using TRIzol reagent (Invitrogen, Carlsbad, CA, USA) according to the manufacturer's protocol and quantified using a NanoDrop 2000 spectrophotometer (Thermo Scientific Inc, Carlsbad, CA, USA). The cDNA was prepared using the PrimerScriptTM RT reagent kit (Takara Clontech, Kyoto, Japan). Real‐time PCR was performed in triplicates using a SuperReal PreMix Plus (SYBR Green) kit (Takara Clontech) on a CFX Connect Real‐Time System (Bio‐Rad, Hercules, CA, USA). Each reaction contained 12.5 μL of 2xSYBR^®^ Primix Ex Taq™ (with SYBR Green I), 300 nmol/L oligonucleotide primers synthesized by Generay Corp. (Nanjing, China) and 1 μL cDNA in a final volume of 25 μL. The thermal cycling conditions included an initial denaturation step at 95°C for 30 seconds, 40 cycles of 95°C for 5 seconds and 60°C for 30 seconds. RNA expression was normalized to levels of β‐actin mRNA. The sequences for RT‐qPCR primers were as follows: human E‐cadherin sense: 5′‐TCACGCTGTGTCATCCAACGG‐3′ and antisense: 5′‐TAGGTGTTCACATCATCGTCCGC‐3′; human α‐SMA sense: 5′‐CAGAAGGAGATCACGGCCCTAG‐3′ and antisense: 5′‐CGGCTTCATCGTATTCCTGTTTG‐3′; human fibronectin sense: 5′‐AAGACCATACCCGCCGAATG‐3′ and antisense: 5′‐GGCATTTGGATTGAGTCCCG‐3′; human β actin sense: 5′‐CATGTACGTTGCTATCCAGGC‐3′ and antisense: 5′‐CTCCTTAATGTCACGCACGAT‐3′.

Quantification of miRNA expression was performed using the miDETECT kit (RiboBio) on a CFX Connect Real‐Time System (Bio‐Rad). SnRNA U6 and Cel‐miR‐39 were used to normalize for technical variation between cell samples and exosome samples, respectively, as previously described.^26^

### 2.11 Western blot analysis

Cells/exosomes were lysed in RIPA buffer (Beyotime, Shanghai, China) supplemented with phenylmethylsulphonyl fluoride and PhoSTOP EASY pack phosphatase inhibitor (Roche, Mannheim, Germany) on ice for 30 m, 48 hours after treatment. The lysates were clarified by centrifugation at 8317 *g* for 5 m at 4°C. Total protein concentration was quantified by a bicinchoninic acid assay kit (Thermo Scientifics), and 40 μg protein was loaded and separated on SDS‐PAGE gels and transferred onto nitrocellulose membrane (Bio‐rad). The membranes were blocked using 5% BSA (Sigma‐Aldrich) in PBS for 45 m at room temperature to prevent non‐specific binding. The membranes were then incubated with primary antibodies diluted in 2% BSA in PBS with 0.1% Tween‐20 (PBS‐T) at 4°C overnight. After rinsing with PBS‐T for three times, the membranes were incubated with IRDye^®^ 680LT Goat anti‐rabbit or IRDye^®^ 800CW Goat anti‐mouse secondary antibodies (Li‐Cor Biosciences) at room temperature for 1 hour. After three washes with PBS‐T, the bound antibody was detected using an Odyssey infrared imaging system (Li‐Cor Biosciences). The band intensities were analysed using Odyssey software and normalized to β‐actin or GAPDH.

### 2.12 Immunofluorescence analysis

Cells were seeded and cultured in a 24‐well plate inlaid with glass coverslips. After treatment, cells were washed and fixed in cold acetone for 5 m. After three washes with PBS, cells were blocked with 2% BSA for 1 hour at room temperature and incubated with the primary antibodies overnight at 4°C. After three rinses with PBS, the coverslips were then incubated with FITC‐conjugated secondary antibodies for 1 hour at room temperature. After counterstaining with 4,6‐diamidino‐2‐phenylindole (DAPI), the stained coverslips were mounted and visualized under a confocal microscope (Carl Zeiss, LSM710, Jena, Germany).

### 2.13 Statistical analysis

All experiments were performed at least three times. The mean and SEM were calculated on all parameters determined in this study. Statistical significance was analysed by one‐way ANOVA or two‐tailed Student's *t* test. A value of *P* < .05 was accepted as statistically significant.

## 3 RESULTS

### 3.1 EMT induction in ARPE‐19 cells

**FIGURE 1:**
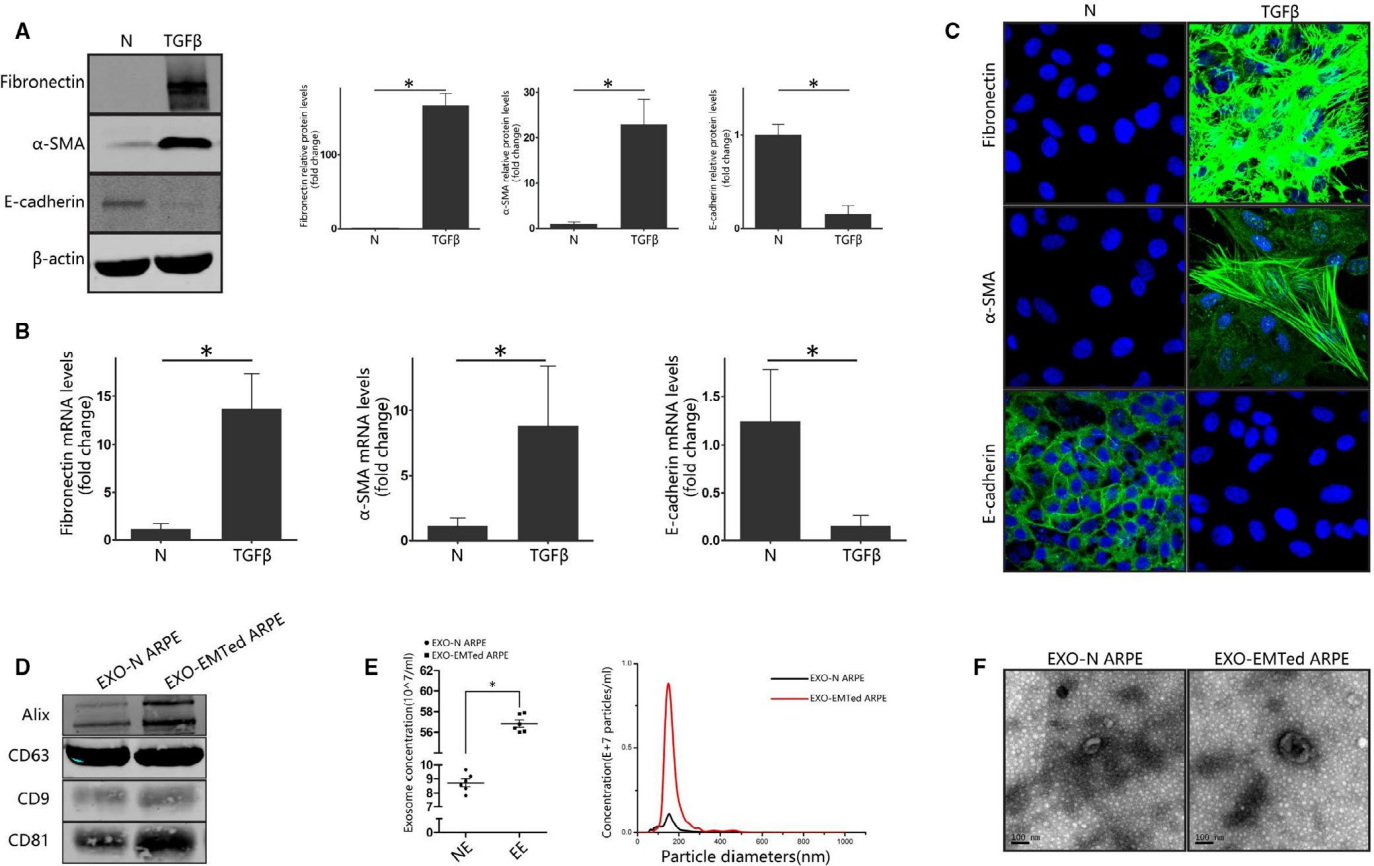
EMT induction in ARPE‐19 cells and characterization of isolated exosomes (A) Western blot analysis. ARPE‐19 cells were treated with 10 ng/mL TGFβ2 for 48 h. The protein expression of E‐cadherin, α‐SMA and fibronectin was detected by Western blot. Relative protein expression (normalized to β‐actin) was quantified in the Western blots based on grey scale values. The data are presented as the mean ± SEM. n = 3. Statistical significance was analysed by two‐tailed Student's *t* test. *P < .05; (B) Real‐time quantitative PCR analysis. ARPE‐19 cells were treated with 10 ng/mL TGFβ2 for 48 h. The mRNA expression levels of EMT‐related proteins were detected with real‐time quantitative PCR. The data are presented as the mean ± SEM, n = 3. Statistical significance was analysed by two‐tailed Student's *t* test *P < .05; (C) Immunofluorescence analysis of EMT‐related proteins in ARPE‐19 cells. After treatment with TGFβ2 for 48 h, EMT‐related proteins were detected using appropriate antibodies. Nuclei were stained with DAPI. The slides were examined by confocal microscopy. Original magnification: 630×, oil. Scale bar: 10 μm; (D) ALIX, CD63, CD9 and CD81 (common exosomal markers) immunoblots of exosomes derived from normal and EMTed ARPE‐19 cells. (E) NTA analysis of exosomes from normal and EMTed ARPE‐19 cells. (F) TEM analysis of exosomes from normal and EMTed ARPE‐19 cells. Scale bar: 100 nm. EXO‐N ARPE, exosome derived from normal ARPE‐19 cells; EXO‐EMTed ARPE, exosome derived from EMTed ARPE‐19 cells; N: control condition

We first used TGFβ2 to induce EMT in ARPE‐19 cells. ARPE‐19 cells were seeded and cultivated for 24 hours. Then, cells were starved using DMEM/F12 medium supplemented with 1% penicillin‐streptomycin without FBS for 24 hours before treatment with TGFβ2. Treatment with 10 ng/mL TGFβ2 for 48 hours significantly reduced the expression of E‐cadherin and increased the expression of α‐SMA and fibronectin at both the mRNA and protein level (Figure 1A,B). These results were also validated by immunofluorescence analysis (Figure 1C). Our results suggested that ARPE‐19 cells went through EMT after 48 hours treatment with 10 ng/mL TGFβ2.

### 3.2 Exosome extraction and identification from EMTed ARPE‐19 cells

Exosomes derived from ARPE‐19 cell supernatants were extracted and characterized by Western blot, TEM and NTA. Exosomal markers (Alix, CD63, CD9, and CD81) were detected by Western blot. Morphological feature of extracted vesicles was observed by TEM, which was consistent with the characteristics of exosomes. The concentration and size distribution of extracted vesicles were analysed by NTA. Peaks vesicle size for both groups was within the expected size of exosomes. The concentration of exosomes from EMTed ARPE‐19 cells was 6.5‐fold higher than that of normal ARPE‐19 cells (Figure 1D‐F).

### 3.3 Exosomes from EMTed ARPE‐19 cells promote further EMT in recipient ARPE‐19 cells

**FIGURE 2:**
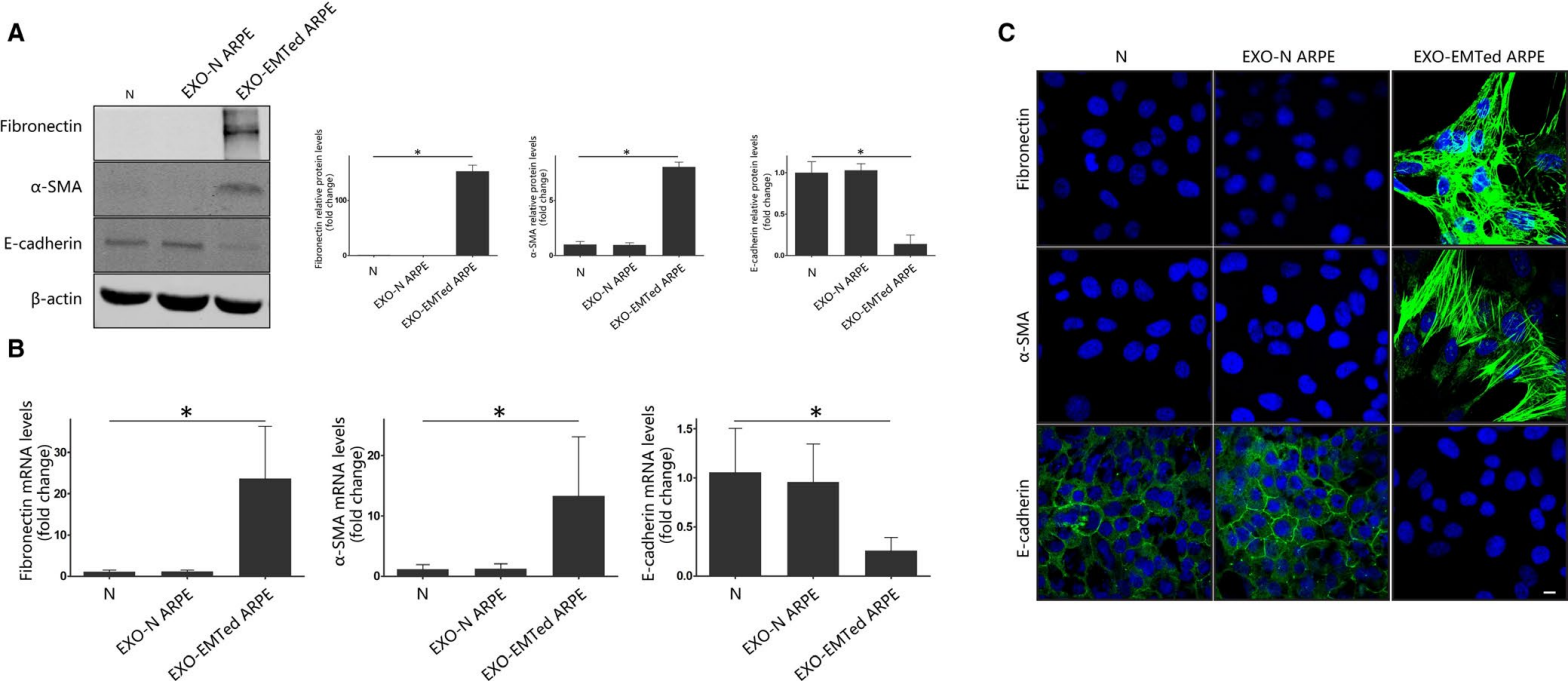
Exosomes from EMTed ARPE‐19 cells induce EMT in recipient ARPE‐19 cells. (A) Western blot analysis. Recipient ARPE‐19 cells were incubated with 100 μg/mL exosomes from EMTed ARPE‐19 cells for 48 h. The protein expression of E‐cadherin, α‐SMA and fibronectin was detected by Western blot. Relative protein expression (normalized to β‐actin) was quantified in Western blots based on grey scale values. The data are presented as the mean ± SEM, n = 3. Statistical significance was analysed by one‐way ANOVA. *P < .05; (B) Real‐time quantitative PCR analysis. Recipient ARPE‐19 cells were incubated with 100 μg/mL exosomes from EMTed ARPE‐19 cells for 48 h. The mRNA expression levels of EMT‐related proteins were detected with real‐time quantitative PCR. The data are presented as the mean ± SEM, n = 3. Statistical significance was analysed by one‐way ANOVA. *P < .05; (C) Immunofluorescence analysis of EMT‐related proteins in ARPE‐19 cells. After incubation with 100 μg/mL exosomes from EMTed ARPE‐19 cells for 48 h, EMT‐related proteins were detected using appropriate antibodies. Nuclei were stained with DAPI. The slides were examined by confocal microscopy. Original magnification: 630×, oil. Scale bar: 10 μm. EXO‐N ARPE, exosome derived from normal ARPE‐19 cells; EXO‐EMTed ARPE, exosome derived from EMTed ARPE‐19 cells; N, control condition

We then examined whether exosomes from EMTed ARPE‐19 cells were able to induce EMT in normal ARPE‐19 cells. We used 100 μg/mL exosomes from both normal and EMTed ARPE‐19 cells to stimulate ARPE‐19 cells. At 48 hours after treatment, protein and mRNA levels of EMT markers were detected by Western blot, RT‐qPCR and immunofluorescence. Treatment with exosomes from EMTed ARPE‐19 cells up‐regulated α‐SMA and fibronectin expression and down‐regulated E‐cadherin expression (Figure 2A‐C).

**FIGURE 3:**
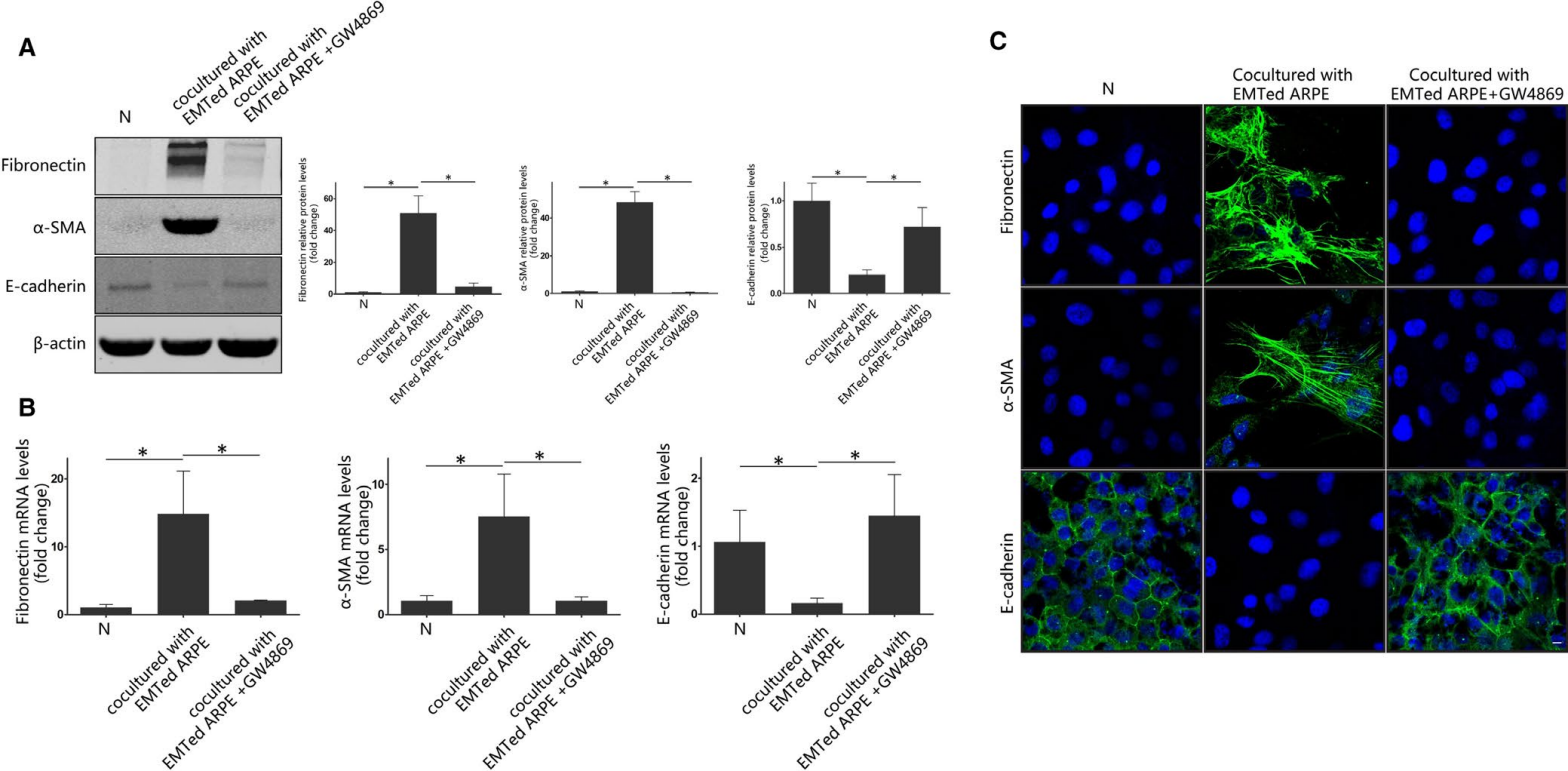
Co‐culture of normal and EMTed ARPE‐19 cells. A, Western blot analysis. Recipient ARPE‐19 cells were co‐cultured with EMTed ARPE‐19 cells with or without GW4869 for 48 h. The protein expression of E‐cadherin, α‐SMA and fibronectin was detected by Western blot. Relative protein expression (normalized to β‐actin) was quantified in Western blots based on grey scale values. The data are presented as the mean ± SEM, n = 3. Statistical significance was analysed by one‐way ANOVA. *P < .05; B, Real‐time quantitative PCR analysis. Recipient ARPE‐19 cells were co‐cultured with EMTed ARPE‐19 cells with or without GW4869 for 48 h. The mRNA expression levels of EMT‐related proteins were detected with real‐time quantitative PCR. The data are presented as the mean ± SEM, n = 3. Statistical significance was analysed by one‐way ANOVA. *P < .05; (C) Immunofluorescence analysis of EMT‐related proteins in recipient ARPE‐19 cells. After co‐culturing with EMTed ARPE‐19 cells for 48 h, EMT‐related proteins were detected using appropriate antibodies. Nuclei were stained with DAPI. The slides were examined by confocal microscopy. Original magnification: 630×, oil. Scale bar: 10 μm

We also conducted co‐culture experiments with EMTed ARPE‐19 cells and normal ARPE‐19 cells, using transwell co‐culture systems. As shown in Figure 3, co‐culturing normal ARPE‐19 cells with EMTed ARPE‐19 cells significantly reduced the expression of E‐cadherin and increase the expression of α‐SMA and fibronectin in the normal cells. However, this effect was abolished when cells were additionally treated with GW4869, an exosome secretion inhibitor (Figure 3A‐C). Our results suggest that exosomes from EMTed ARPE‐19 cells promote EMT in normal ARPE‐19 cells.

### 3.4 Verification of exosome internalization

**FIGURE 4:**
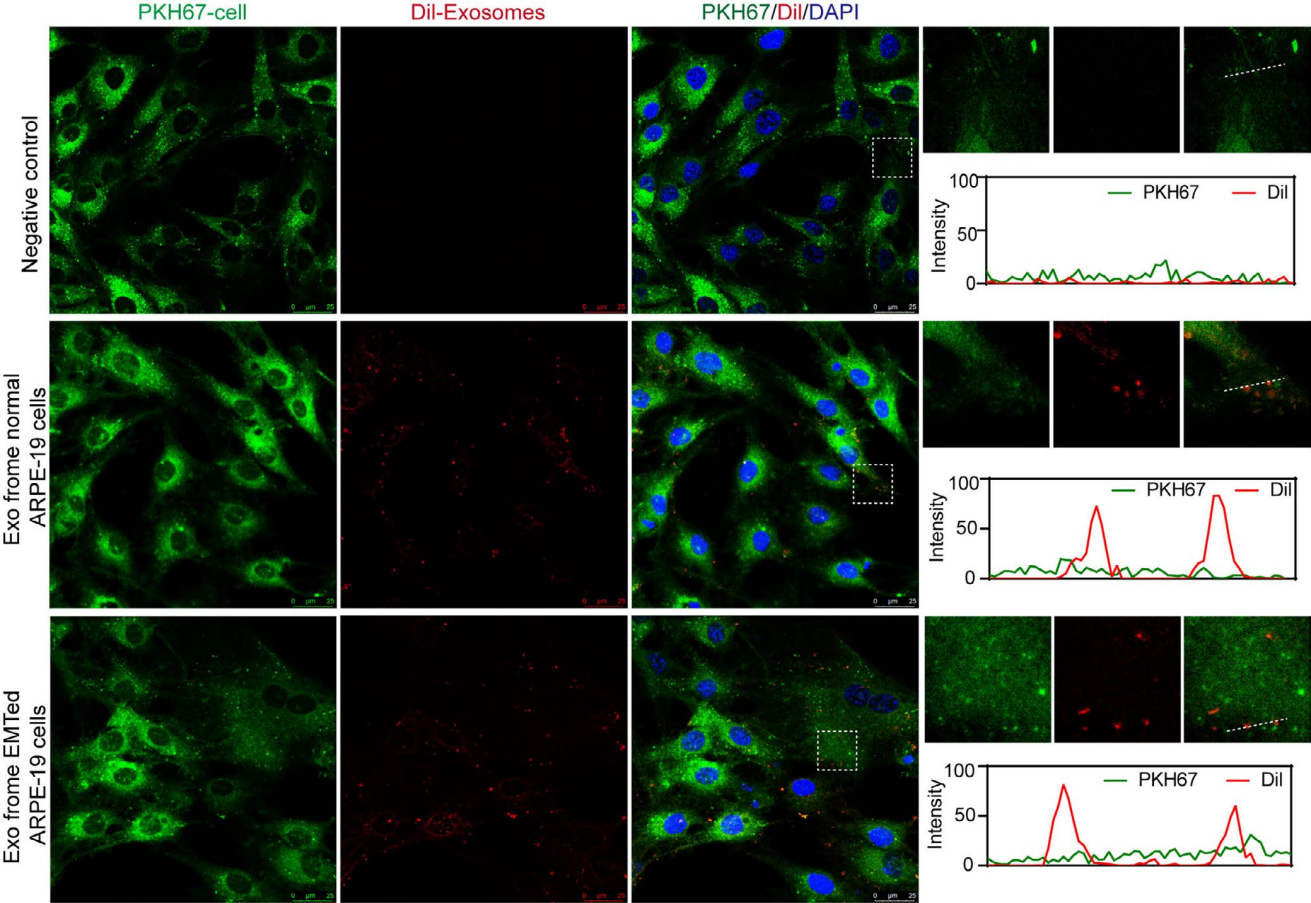
Detection of exosome uptake by recipient ARPE‐19 cells. Recipient ARPE‐19 cells that had been incubated for 24 h with CM‐Dil‐labelled exosomes are depicted (CM‐Dil in red, PKH in green, DAPI in blue). Scale bar: 25 μm. Images shown on the right were magnified from the white boxes shown in images on the left. Profile analysis was performed using Image J software

To verify that ARPE‐19 cells can internalize exosomes, CM‐Dil‐labelled exosomes were incubated with PKH67‐labelled ARPE‐19 cells for 24 hours, and the cellular uptake of exosomes was imaged via confocal microscopy. The internalization of exosomes was confirmed visually by the presence of intracellular punctate fluorescence in the cytoplasm of the cells. There were no differences in the uptake of normal ARPE‐19‐derived exosomes and EMTed ARPE‐19‐derived exosomes (Figure 4).

### 3.5 miRNA expression profile of EMTed ARPE‐19 cells‐derived exosomes

**FIGURE 5:**
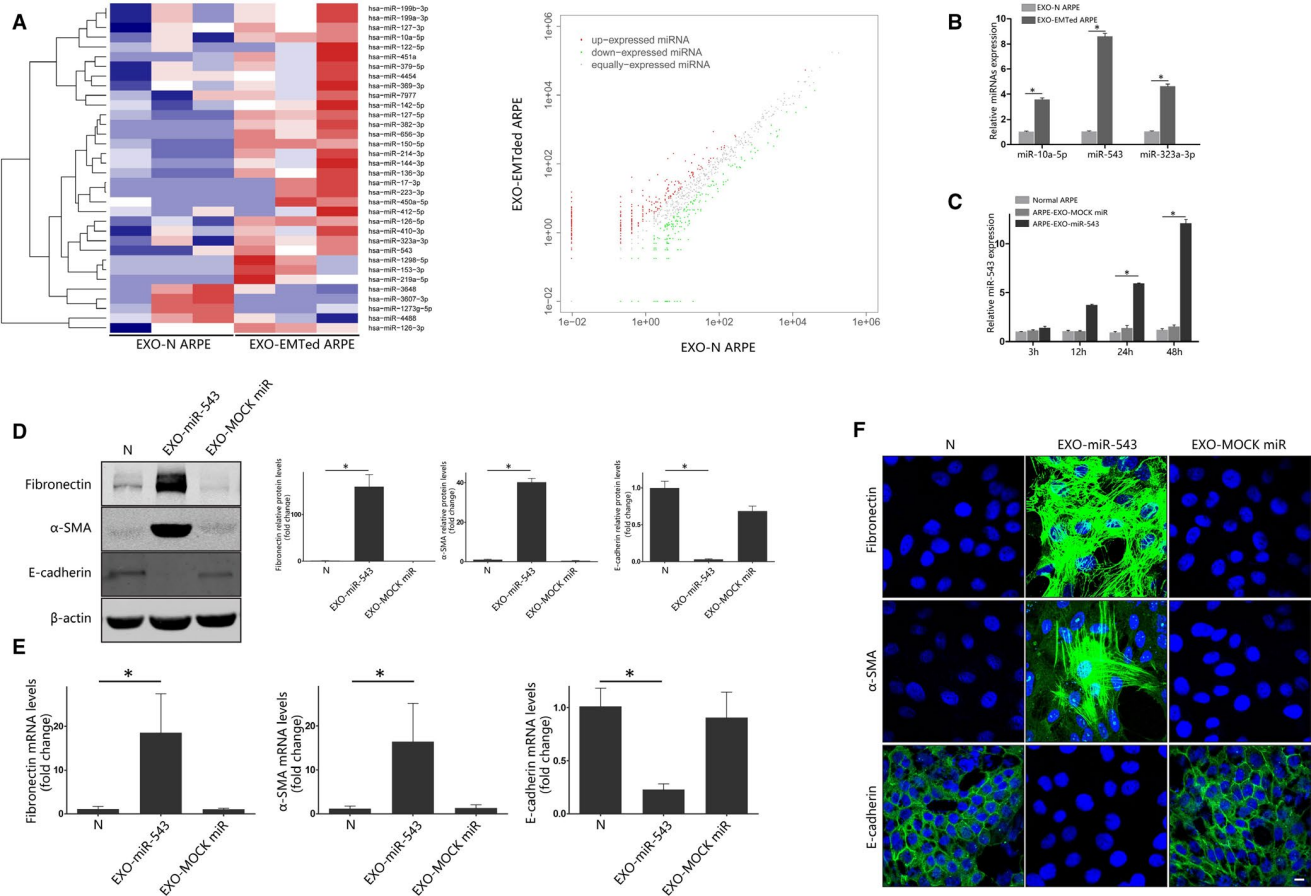
Exosomal microRNA sequencing and exosomal miR‐543 promote EMT of ARPE‐19 cells. A, Heat map of differentially expressed exosomal miRNA from normal and EMTed ARPE‐19 cells. B, Real‐time quantitative PCR verification of miRNA‐seq. Exosomal miR‐10a‐5P, miR‐543 and miR‐323a‐3p expression in normal and EMTed ARPE‐19 cells was detected by RT‐qPCR. n = 3, Statistical significance was analysed by two‐tailed Student's *t* test. *P < .05; (C) Real‐time quantitative PCR analysis of miR‐543 in recipient ARPE‐19 cells. ARPE‐19 cells were incubated with 100 μg/mL exosome loaded with miR‐543 mimics or miRNA mimics control for 3, 12, 24 and 48 h, and the cellular miR‐543 levels were detected with RT‐qPCR. Statistical significance was analysed by one‐way ANOVA. D, Western blot analysis. Recipient ARPE‐19 cells were incubated with 100 μg/mL exosomes loading with miR‐543 mimics or miRNA mimics control for 48 h. The protein expression of E‐cadherin, α‐SMA and fibronectin was detected with Western Blot. Relative protein expression (normalized to β actin) was quantified in the Western blots based on their grey scale values. The data are presented as the mean ± SEM. n = 3. Statistical significance was analysed by one‐way ANOVA. *P < .05; E, Real‐time quantitative PCR analysis. Recipient ARPE‐19 cells were incubated with 100 μg/mL exosomes loaded with miR‐543 mimics or miRNA mimics control for 48 h. The mRNA expression levels of EMT‐related proteins were detected with real‐time quantitative PCR. The data are presented as the mean ± SEM, n = 3. Statistical significance was analysed by one‐way ANOVA. *P < .05; F, Immunofluorescence analysis of EMT‐related proteins in ARPE‐19 cells. After incubation with 100 μg/mL exosomes loaded with miR‐543 mimics or miRNA mimics control for 48 h, EMT‐related proteins were detected using appropriate antibodies. Nuclei were stained with DAPI. The slides were examined by confocal microscopy. Original magnification: 630×, oil. Scale bar: 10 μm. EXO‐N ARPE: exosome derived from normal ARPE‐19 cells；EXO‐EMTed ARPE: exosome derived from EMTed ARPE‐19 cells; EXO‐miR‐543: exosome loaded with miR‐543 mimics; EXO‐ MOCK miR: exosome loaded with miRNA mimics control

MiRNA expression profiles of exosomes from normal ARPE‐19 cells and EMTed ARPE‐19 cells were examined via Illumina HiSeq 2500 high‐throughput sequencing. We compared the expression levels of miRNAs in exosomes from normal ARPE‐19 and EMTed ARPE‐19 cells. Using a two‐fold change and *P* < .05 as the threshold cut‐off values, 34 miRNAs were significantly different between normal ARPE‐19 and EMTed ARPE‐19 cells exosomes. Among the differentially expressed miRNAs, 30 miRNAs were up‐regulated in EMTed ARPE‐19 cells exosomes compared with normal ARPE‐19 cells while 4 were down‐regulated (Figure 5A). Among the 34 miRNAs, miR‐10a‐5p, miR‐543 and miR‐323a‐3p have been previously reported as playing a role in the EMT of other cell types or tissues. The exosomal levels of these three miRNAs were measured by RT‐qPCR to validate our miRNA‐seq results. Consistent with miRNA‐seq, RT‐qPCR showed that exosomes derived from EMTed ARPE‐19 cells had significantly increased miR‐10a‐5p, miR‐543 and miR‐323a‐3p levels, compared with those derived from normal ARPE‐19 cells (Figure 5B).

### 3.6 Exosomal miR‐543 induces EMT in ARPE‐19 cells

We selected miR‐543 for further study, as it was significantly increased in exosomes from EMTed ARPE‐19 cells, and was the most highly expressed miRNA (Figure 5B). In addition, several studies have suggested that miR‐543 plays a role in EMT in a variety of cell types.^27^, ^28^, ^29^, ^30^, ^31^ To test whether exosomal miR‐543 could induce EMT of normal ARPE‐19 cells, miR‐543 mimics and control miRNA mimics were separately transfected into exosomes derived from normal ARPE‐19 cells. Then, two groups of these exosomes were applied to stimulated recipient ARPE‐19 cells. RT‐qPCR results showed that after being treated with miR‐543 mimic‐loaded exosomes for 48 hours, significant miR‐543 levels were detected in recipient ARPE‐19 cells (Figure 5C). At the same time point, exosomes transfected with miR‐543 mimics significantly increased the expression of α‐SMA and fibronectin, and decreased the expression of E‐cadherin in normal ARPE‐19 cells. No significant changes in levels of these three proteins were observed in ARPE‐19 cells treated with exosome transfected with miRNA mimic controls (Figure 5D‐F). These results suggest that exosomal miR‐543 induces EMT in ARPE‐19 cells.

## 4 DISCUSSION

Proliferative vitreoretinopathy, which is a severe blinding complication that usually occurs after RRD pre‐ or post‐operatively, is characterized by the cellular proliferation and formation of sub/epiretinal fibrotic membranes. Subsequent traction of these membranes leads to detachment of the retina and irreversible visual impairment.^4^ Based on pathology studies of the PVR membrane, researchers have found that RPE‐derived cells represent the largest cellular component of the membrane. RPE cells are thought to play a primary role in the pathogenesis of PVR, and the transformation process of RPE cells was recently shown to be mediated by EMT.^32^ Since then, EMT of RPE cells has been regarded as the trigger point in the pathogenesis of PVR.

Exosomes, a set of nano‐sized vesicles ranging 30‐150 nm in diameter, are secreted by most eukaryotic and prokaryotic cells. The secretion and contents of exosomes vary by cell type and cell status. As a cell‐cell communication mechanism, exosomes can be secreted from donor cells into the extracellular environment, and then internalized by recipient cells via blood transport or direct spreading. It has been shown that exosomes have fundamental biological roles in the regulation of normal physiological and pathological processes such as EMT. For example, He et al found that exosomal miR‐499a‐5p promotes EMT via the mTOR signalling pathway in lung adenocarcinoma.^33^ Another study conducted by Wang et al suggested that exosomes can promote EMT of clear cell renal cell carcinoma via remote miR‐19b‐3p.^34^ Exosomes were also demonstrated to mediate paracrine miR‐34a‐5p expression, and to induce EMT to promote cancer cell metastasis in oral cancer cells.^23^

**FIGURE 6:**
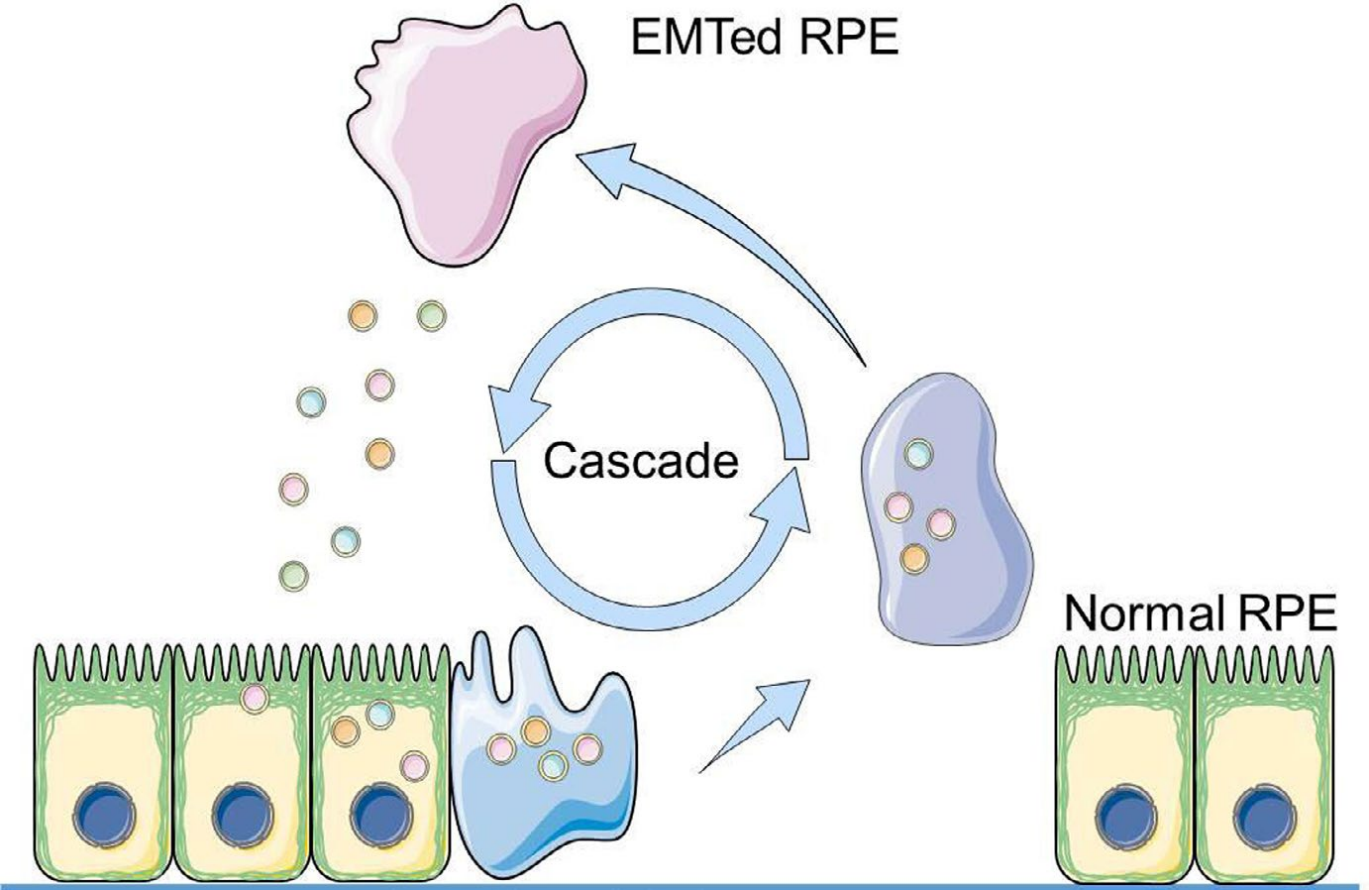
Mechanism of EMT cascade mediated by exosome. In the PVR microenvironment, EMT allows RPE cells to transdifferentiate into mesenchymal cells and secrete exosomes containing pro‐EMT ingredients, including miR‐543. These pathologic exosomes are adopted by healthy RPE cells and a second round of RPE EMT starts. This EMT cascade causes more and more RPE cells to transdifferentiate into myofibroblasts, participate in the formation of PVR membrane, and eventually leads to the rapid progression of PVR

Current understanding of PVR pathogenesis cannot explain the origin and mechanism of action of massive RPE cells in PVR membrane. Although some RPE cells may dissociate in RRD and have been found in the vitreous fluid of RRD patients, the number of these RPE cells is insufficient to form a PVR membrane. The formation of the PVR membrane likely reflects a complicated process in the vitreoretinal microenvironment, as exosomes do not exist in this environment normally. Based on this fact, we have been suggested that exosomes may mediate the EMT cascade of RPE cells, and that this cascade effect causes an exponential increase in RPE EMT. Eventually, massive numbers of previously healthy RPE became affected, transdifferentiate, and grown into fibrotic membranes that lead to PVR (Figure 6).

In this study, we first investigated the effect of EMT on the secretion of exosomes from ARPE‐19 cells. We use TGFβ to obtain EMTed ARPE‐19 cells and collect exosomes for subsequent experiments. TGFβ, a classic EMT inducer that is used in many cell types, was also found in the vitreous fluid of PVR patients.^35^ Our group has utilized TGFβ to induce EMT in RPE cells since 2011.^36^ Consistent with previous studies, 48 hours treatment with TGFβ resulted in obvious changes to both epithelial and mesenchymal marker proteins in ARPE‐19 cells.^36^, ^37^, ^38^, ^39^, ^40^

We next extracted exosomes from both normal ARPE‐19 cells and EMTed ARPE‐19 cells. For exosome extraction, many methods have been previously used, including differential ultracentrifugation,^41^ PEG precipitation,^42^ sucrose and iodixanol ultracentrifugation,^43^ immunoaffinity capture^44^ and size‐exclusion chromatography.^45^ Of these, differential centrifugation and PEG precipitation are the two methods that are most commonly used. The deposit from both methods often contains lipoprotein particles and large protein aggregates. As the classic exosome extraction protocol, differential centrifugation shows better purity, higher efficiency and lower cost compared with other methods. In addition, PEG precipitation has been previously used to separate viruses and has been shown to be a viable method for exosome extraction in recent years.^42^, ^46^, ^47^ PEG precipitation method‐based kits such as ExoQuick and Total Exosome Isolation reagent have also become increasingly popular due to their ease of use and improved protection of exosome contents.^48^, ^49^ In our experiments, PEG precipitation was adopted because of its efficiency and convenience in dealing with large volumes of supernatant.

The concentration and content of secreted exosomes vary by cell type and cell status. Previous studies have suggested that massive exosome release from cells underlies specific biologic processes. Research conducted by Wang et al suggested that breast cancer cells secrete much more exosomes when cultured under hypoxic conditions.^50^ Another study suggested that LPS, an EMT inducer, can significantly increase the release of exosome from macrophages.^51^ Wang et al also observed massive exosome release from astrocytes upon treatment with TNFα.^52^ Consistent with previous reports, our results suggested that ARPE‐19 cells secreted massive amounts of exosomes when EMT was induced by TGFβ‐2. As for exosomal contents, previous research compared the protein differences between exosomes from normal MDCK and EMTed MDCK cells and found significantly different levels of EMT‐related proteins such as E‐cadherin, MMP and integrin between the two groups.^53^ Another study conducted by Tang et al suggested that the function and miRNA profile of exosomes change after EMT in human lung cancer cells.^54^

In this study, we focused on the miRNA profile of exosomes from RPE of both groups. MiRNAs have been shown to play pivotal roles in the regulation of EMT in a variety of tissues and organs.^55^ Among ophthalmologic research, several studies have also highlighted the role of miRNA in EMT of RPE. Specifically, Takayama et al found that miR148a significantly increases in cases of retinal detachment and showed that miR148a promotes EMT in RPE cells.^56^ Another study conducted by Chen et al suggested that the miRNA expression profile changes in RPE cells after the induction of EMT by TGFβ‐2.^57^ Jun et al also demonstrated that miR124 was down‐regulated after TGFβ‐1 treatment, and that overexpression of miR124 repressed the TGFβ‐1‐induced EMT of RPE by targeting RHOG.^58^ The role of exosomal miRNAs has also been investigated. A study conducted by Li et al suggested that CAFs contribute to oral cancer cell proliferation and metastasis via exosome miR‐34a‐5p.^23^ In addition, Ota et al found that exosomal miR‐30e suppresses cell invasion and migration via inhibiting the EMT of cholangiocarcinoma cells.^59^ However, the effect of exosomal miRNAs in the EMT of RPE has not yet been reported.

Our miRNA‐seq results showed that 34 miRNAs were significantly different between normal ARPE‐19 cell‐ and EMTed ARPE‐19 cell‐derived exosomes. Among the differentially expressed miRNAs, 30 miRNAs were up‐regulated in exosomes from EMTed ARPE‐19 cells, while 4 were down‐regulated.

In the study mentioned above,^53^ exosomes from EMTed cells were speculated to further promote EMT; however, no functional analyses were conducted.^60^ In our study, we incubated recipient ARPE‐19 cells with exosomes to investigate the effect of exosomes in the EMT of ARPE‐19 cells. Our results showed that exosomes derived from EMTed ARPE‐19 cells could induce the up‐regulation of mesenchymal proteins and the down‐regulation of epithelial proteins. Our results suggest that there is a pro‐EMT effect of exosomes released from EMTed ARPE‐19 cells. MiRNA‐seq was applied to screen the EMT‐related miRNAs in exosomes from two groups, and 34 differential expressed miRNAs were identified as a result. Three of the 34 miRNAs were previously reported to play a role in EMT: miR‐10a‐5p, miR‐543 and miR‐323a‐3p. Of these, miR‐543 has been reported to promote cell migration and invasion by targeting SPOP in gastric cancer.^61^ Another study conducted by Zhao et al suggested that miR‐543 promotes migrant, invasion and EMT in oesophageal cancer cells.^27^ MiR‐543 has also been shown to promote EMT in prostate cancer, via targeting RKIP.^31^ In our study, miRNA‐543 mimics were transfected into exosomes, and these exosomes were incubated with recipient ARPE‐19 cells. Up‐regulation of mesenchymal proteins and down‐regulation of epithelial proteins were found in recipient ARPE‐19 cells. These results suggest that exosomal miR‐543 induces EMT in ARPE‐19 cells, and that the EMT cascade process can be at least partially completed by exosomal miR‐543.

In PVR pathology, the disease progresses from several RPE cells floating within the vitreous at disease onset to the formation of a PVR membrane containing huge amounts transdifferentiated RPE cells, suggesting that more and more RPE cells go through EMT as the disease progress. In the local vitreoretinal environment, EMTed RPE cells may secrete exosomes loaded with pro‐EMT cargo. These exosomes may affect in situ RPE cells and promote a second round of RPE EMT. This EMT cascade causes increasing numbers of RPE cells to transdifferentiate into myofibroblasts, participate in the formation of the PVR membrane, and eventually leads to the rapid progression of PVR.

As a classic RPE cell line, ARPE‐19 cells were widely used to study the physiology and function of RPE cells in vitro. Recently, primary RPE cells (human primary RPE/human foetal RPE) have been recommended as a superior model for the study of RPE due to their improved barrier function, cellular morphology and RPE‐related marker expressions. However, the huge amount of cells needed for the isolation of primary RPE cell‐derived exosomes limited their usage in this study. Another limitation of our work is that we focused on the effects of miRNAs from exosomes, while there are many other components within exosomes. Future studies will be needed to explore their effects on PVR.

## 5 CONCLUSION

Our preliminary study suggests that ARPE‐19 cells secret massive amounts of exosomes after EMT, and that the exosomes from EMTed ARPE‐19 cells induce further EMT of recipient ARPE‐19 cells. This EMT cascade mediated by exosomes could be at least partially accounted for by exosomal miR‐543. Primary RPE and animal models of PVR may also be adopted to confirm the results derived from ARPE‐19 in the future. Moreover, the comprehensive and precise role of exosomes in the pathogenesis of PVR and the detailed underlying mechanisms still need further investigation.

## CONFLICT OF INTEREST

The authors declare that they have no competing interests.

## AUTHOR CONTRIBUTION

**Yao Zhang:** Conceptualization (lead); Data curation (lead); Investigation (lead); Methodology (lead); Project administration (lead); Software (lead); Writing‐original draft (lead); Writing‐review & editing (equal). **Kaizhe Wang:** Conceptualization (lead); Data curation (lead); Investigation (lead); Methodology (lead); Project administration (lead); Software (lead); Writing‐original draft (lead); Writing‐review & editing (equal). **Jiabin Pan:** Investigation (equal); Software (equal). **Shuai Yang:** Methodology (equal). **Haipei Yao:** Methodology (equal). **Min Li:** Methodology (equal). **Hui Li:** Methodology (equal). **Hetian Lei:** Methodology (equal). **Haiying Jin:** Project administration (lead); Supervision (lead); Validation (lead); Writing‐review & editing (lead). **Fang Wang:** Funding acquisition (lead); Project administration (lead); Supervision (lead); Validation (lead); Writing‐review & editing (lead).

## ETHICAL APPROVAL

This study was approved by the Science and Technology Commission of Shanghai Municipality (ID: SYXK 2011‐0111). All methods were performed in accordance with the relevant guidelines and regulations.

## Data Availability

The data that support the finding of this study are available from the corresponding author upon reasonable request.
